# The use and protective effects of transcutaneous electrical acupoint stimulation during abdominal surgery: study protocol for a multicenter randomized parallel controlled trial

**DOI:** 10.1186/s13063-019-3558-2

**Published:** 2019-07-29

**Authors:** Yuantao Hou, Qi Yan, Haiyan An, Junlu Wang, Ming Tian, Wensheng Zhao, Anshi Wu, Yi Feng

**Affiliations:** 1 Peking University People’s Hospital, Beijing, China; 2 First Affiliated Hospital of Wenzhou Medical University, Wenzhou, China; 3 Zhejiang Province Wencheng County People’s Hospital, Wenzhou, China; 4 Beijing Friendship Hospital, Capital Medical University, Beijing, China; 5 Zhejiang Integrated Traditional and Western Medicine Hospital, Hangzhou, China; 6 Beijing Chaoyang Hospital, Capital Medical University, Beijing, China

**Keywords:** Transcutaneous electrical acupoint stimulation, Anesthesia management, Gastrointestinal surgery, Organ protection

## Abstract

**Background:**

Acupuncture-balanced anesthesia has been found to offer protective benefits. Electrical stimulation at certain acupoints can potentially promote perioperative gastrointestinal function recovery. The purpose of this study is to explore the effects of acupuncture-balanced anesthesia on the postoperative recovery of gastrointestinal function, on anesthesia strategies for abdominal surgery, on postoperative pain treatment, and on any associated complications or alterations in immune function. We further seek to verify the protective effects of transcutaneous electrical acupoint stimulation (TEAS), to explore possible underlying neuroimmune–endocrine mechanisms, and to thereby develop an optimized acupuncture-balanced anesthesia strategy suitable for abdominal surgery. Together, these findings will provide a scientific basis for the clinical utilization of acupuncture-balanced anesthesia in the context of abdominal surgery.

**Methods/design:**

This study is a multicenter, large-sample, randomized placebo-controlled trial. All subjects will be patients undergoing elective gastric or colorectal surgery. In Part 1, these patients will be stratified according to surgical site (gastric or colorectal), and randomly divided into four groups based on different perioperative interventions: Con group, which will undergo sham TEAS before, during, and after surgery; T1 group, which will receive TEAS during the preoperative and intraoperative periods, and sham TEAS during the postoperative period; T2 group, which will receive TEAS during the preoperative period, sham TEAS during the intraoperative period, and TEAS during the postoperative period; and T3 group, which will receive TEAS before, during, and after operation. Part 2 of this study will focus solely on colorectal surgery patients. All patients will receive TEAS during the preoperative and intraoperative periods, and they will be randomized into four groups according to different postoperative treatments: Con′ group, which will not receive TEAS; T1′ group, which will receive sham TEAS; T2′ group, which will receive 5-Hz TEAS; and T3′ group, which will receive 100-Hz TEAS. Venous blood (5 ml) will be used to measure immunological and inflammatory indexes both at the preoperative stage prior to TEAS and 4–5 days after operation. The primary outcome will be the time to first bowel sounds after surgery. Secondary outcomes will include gastrointestinal functional recovery, analgesic efficacy during the postoperative period, acupuncture-balanced anesthesia efficacy, postoperative nausea and vomiting, and postoperative complications.

**Discussion:**

This study is designed to investigate the clinical value of TEAS during various perioperative periods in those undergoing abdominal surgery, with the overall goal of evaluating the clinical value and advantages of acupuncture-balanced anesthesia, and of providing new strategies for improving patient prognoses.

**Trial registration:**

Chinese Clinical Trial Registry, ChiCTR-TRC-14004435. Registered on 26 March 2014.

**Electronic supplementary material:**

The online version of this article (10.1186/s13063-019-3558-2) contains supplementary material, which is available to authorized users.

## Background

The clinical application of acupuncture and the scientific basis for acupuncture are of increasing interest, as evidenced by a rapid increase in the number of acupuncture-related studies over the past decade. Acupuncture is a form of traditional Chinese medicine, with acupuncture analgesia being one of the most studied and best understood varieties [1]. Despite previous research, studies to date on the protective benefits of acupuncture have failed to properly assess long-term patient prognosis. As such, well-controlled trials exploring these long-term effects have the potential to help decrease medical costs of gastrointestinal surgery and reduce perioperative complications.

At present, acupuncture-related technologies include manual acupuncture, electroacupuncture, and transcutaneous electrical acupoint stimulation (TEAS). As electroacupuncture and TEAS can allow for accurate control of stimulation parameters, they are helpful for quantitative research and are thus favored by acupuncturists. For these reasons, we applied TEAS for use in our study.

Acupuncture-balanced anesthesia is an approach which combines acupuncture with anesthetics. It can significantly enhance the analgesic effect and reduce the dosage of anesthetics [2, 3], and can offer certain beneficial effects, such as alleviating preoperative anxiety [4] and reducing postoperative pain [5] and postoperative nausea and vomiting (PONV) [6]. By lowering the need for anesthetic usage, TEAS may thus allow for better intraoperative circulatory and respiratory stabilization [7], enhanced surgery recovery, reduced complication rates, and better organ functionality [8].

Acupuncture-balanced anesthesia has been found to help protect the brain [9, 10], heart [11], and other tissues, with positive benefits for gastrointestinal function. Acupuncture can regulate vasoactive intestinal peptide levels and improve gastrointestinal motility by relaxing the gastrointestinal smooth muscle [12]. Other studies have found that acupuncture can be helpful as a means of restoring gastrointestinal barrier function [13], and electroacupuncture pretreatment can decrease acetylsalicylic acid-induced ulcer severity by decreasing gastric acidity and increasing cyclooxygenase (COX)-1 and COX-2 expression [14]. The brain–gut axis is a complex network that maintains gastrointestinal homeostasis [15]. Fang et al. [16] found that acupuncture can relieve symptoms of functional dyspepsia, normalizing the function of the brain–gut axis as it pertains to this disease.

A series of animal experiments have revealed that acupuncture can further modulate the regulation of cellular and humoral immunity [17, 18], balancing Th1/Th2/Th17/Treg Th cell subset responses [19] and potentially improving mucosal immune protection by increasing immunoglobulin A secretion [20].

Acupuncture-balanced anesthesia can further reduce stress responses in elderly patients undergoing laparoscopic cholecystectomy, improving their hemodynamic stability [21].

Despite this previous evidence, the majority of studies of acupuncture-balanced anesthesia today have been of a single-center design, and have not assessed relevant clinical prognostic indicators. In addition, most of these acupuncture studies have been limited to the intraoperative period, without fully assessing benefits during the perioperative period. Therefore, there are many vital research questions pertaining to the effects of acupuncture-balanced anesthesia on organ protection that remain to be answered, including the following: whether acupuncture can improve the general condition of patients during the preoperative period (e.g., by alleviating anxiety and pain); whether continuous acupuncture treatment is beneficial for patients’ postoperative recovery, including gastrointestinal function recovery, postoperative pain management, PONV, and postoperative complications; and how perioperative acupuncture influences long-term prognosis, including quality of life and mortality. By studying these key unknown questions, it would be possible to develop novel breakthroughs and extend the clinical application of acupuncture-balanced anesthesia.

## Methods/design

### Design

This multicenter, large-sample, randomized placebo-controlled trial will be conducted in five hospitals at four institutions. The study protocol has been approved by the ethics committee at Peking University People’s Hospital. Patients undergoing elective gastric or colorectal surgery will be selected. Informed consent will be obtained before randomization. This protocol can only be amended with approval from the ethics committee, leading to an appropriate update in the Chinese Clinical Trial Registry. The findings of the study will be disseminated in academic journals and conference presentations.

### Patient population and methodology

#### Inclusion criteria

Patient inclusion criteria for this ongoing trial are as follows: age 18–75 years; consecutive patients undergoing elective abdominal surgery; body mass index 18–31 kg/m^2^; American Society of Anesthesiologists grade I–III; and signed informed consent.

#### Exclusion criteria

Patients meeting any of the following criteria will be excluded from this trial: scars or sensory impairments around the acupoints of interest; infections around the acupoints of interest; peripheral nerve injuries; history of spinal surgery; participation in other clinical trials within the past 4 weeks; inability to understand Numeric Rating Scales (NRS) scores or rejection of patient-controlled intravenous analgesia (PCIA); patients with a cardiac pacemaker; females in periods of menstruation, pregnancy, or breastfeeding; patients with preoperative pain or a history of analgesic usage; patients with mental or neurological diseases; patients who require an intraoperative colostomy; heavy drinking, defined by the National Institute on Alcohol Abuse and Alcoholism as more than three standard drinks (containing 14 g of pure alcohol) per day for women and more than four standard drinks per day for men [22]; patients returning to the intensive care unit; patients with allergies or a history of steroid usage; patients who develop serious intraoperative complications will be excluded after operation (such as blood loss > 2000 ml; brain, heart, and lung-related complications); patients with a history of liver or kidney disease; serum K^+^ > 5.5 mmol/L or < 3.0 mmol/L; preoperative hemoglobin < 7 g/dl; or patients deemed unsuitable by the researchers.

### Randomization and blinding

A random allocation sequence will be implemented using a remote network electronic randomization system (CIMS® Brightechm, Chengdu, China), according to preset conditions and procedures, thus ensuring correct randomization. Random codes will be generated by an independent statistician using a stratified block randomization scheme. Surgical site (gastric or colorectal) will be considered a stratifying factor. Participants will be randomly assigned to each group based on their sequence of visitation to the study centers.

Allocations of subjects will be kept confidential, without the possibility of unblinding except in emergencies and with approval from the principal investigator as a result of serious adverse events in patients. After unblinding, patient trial treatment will be discontinued, and the results of the treatment will be notified to the inspector, with the details, dates, and appropriate signatures being included in a case report form (CRF).

The investigators will be responsible for enrolling patients. Once a patient is successfully enrolled, the practitioners will register and randomize the patient in the randomization system. The practitioners will be aware of the allocation in order to provide treatment for the participants, while the participants, outcome assessors, and data analysts will be blinded to the treatment allocation. Practitioners and outcome assessors will not communicate with each other.

Sham TEAS will be set to 1 mA lower than the current that participants are able to just feel. This is a very strict and quantifiable “minimal stimulus” method in acupuncture-related research [23], and there are published articles supporting this design [24, 25]. In order to maximize participants’ blinding, the same stimulators, electrodes, and acupoints will be selected. The stimulators will be turned on, and a 30-min countdown will be set up for all participants. We will tell all participants that treatment parameters vary on an individual basis, and as such they may have different feelings regarding the treatment.

### Interventions

**Fig. 1:**
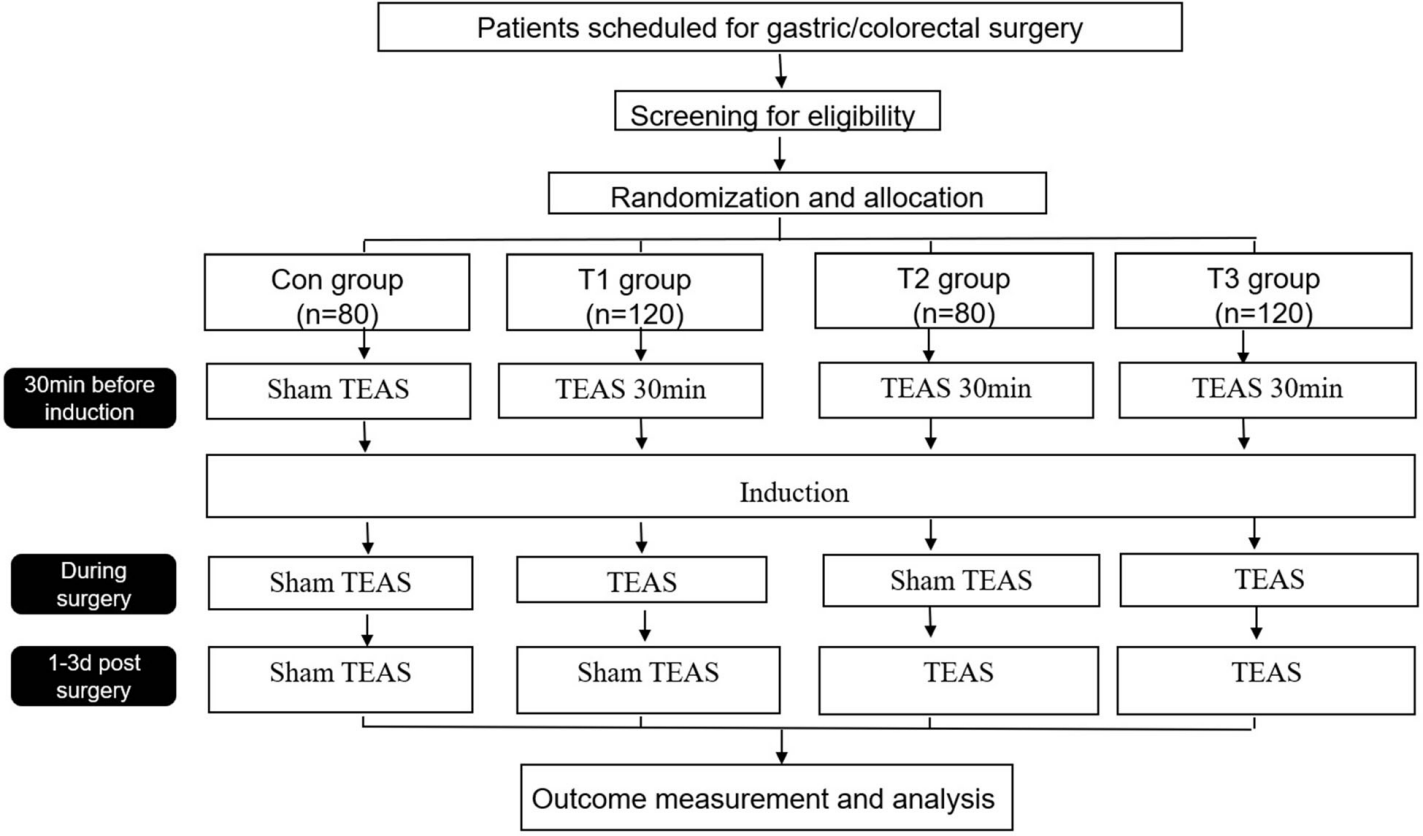
Part 1 trial flowchart. For group details, see Table 1. d days, TEAS transcutaneous electrical acupoint stimulation

For this study, the bilateral Hegu (LI4), Neiguan (P6), Zusanli (ST36), and Sanyinjiao (SP6) acupoints will be selected. A HANS 100B (four conductors, eight electrodes) transdermal acupoint electrical stimulation apparatus (Nanjing Jisheng Company) will be used to stimulate the bilateral Hegu (LI4) and Neiguan (P6) acupoints for 30 min before anesthetic induction and during the intraoperative period until suturing of the surgical site is completed. The Hegu (LI4), Neiguan (P6), Zusanli (ST36), and Sanyinjiao (SP6) acupoints will be stimulated at 9:00 a.m. and 3:00 p.m. each day on the 1st, 2nd, and 3rd postoperative days. Each treatment will last for 30 min. Stimulation parameters will be as follows: a dense-disperse frequency of 2/100 Hz, with true stimulation being the maximum tolerable setting for a patient, and with sham simulation being 1 mA lower than the current that patients are able to just feel. The test groups for Part 1 are presented in Table 1, and a study design flowchart is shown in Fig. 1.

**Table 1:** Part 1 test groups

Group	Sample size (*n*)	Intervention
Con	80	Sham TEAS before, during, and after operation
T1	120	Preoperative and intraoperative TEAS, and postoperative sham TEAS
T2	80	Preoperative TEAS, intraoperative sham TEAS, and postoperative TEAS
T3	120	TEAS before, during, and after operation

*TEAS* transcutaneous electrical acupoint stimulation

**Fig. 2:**
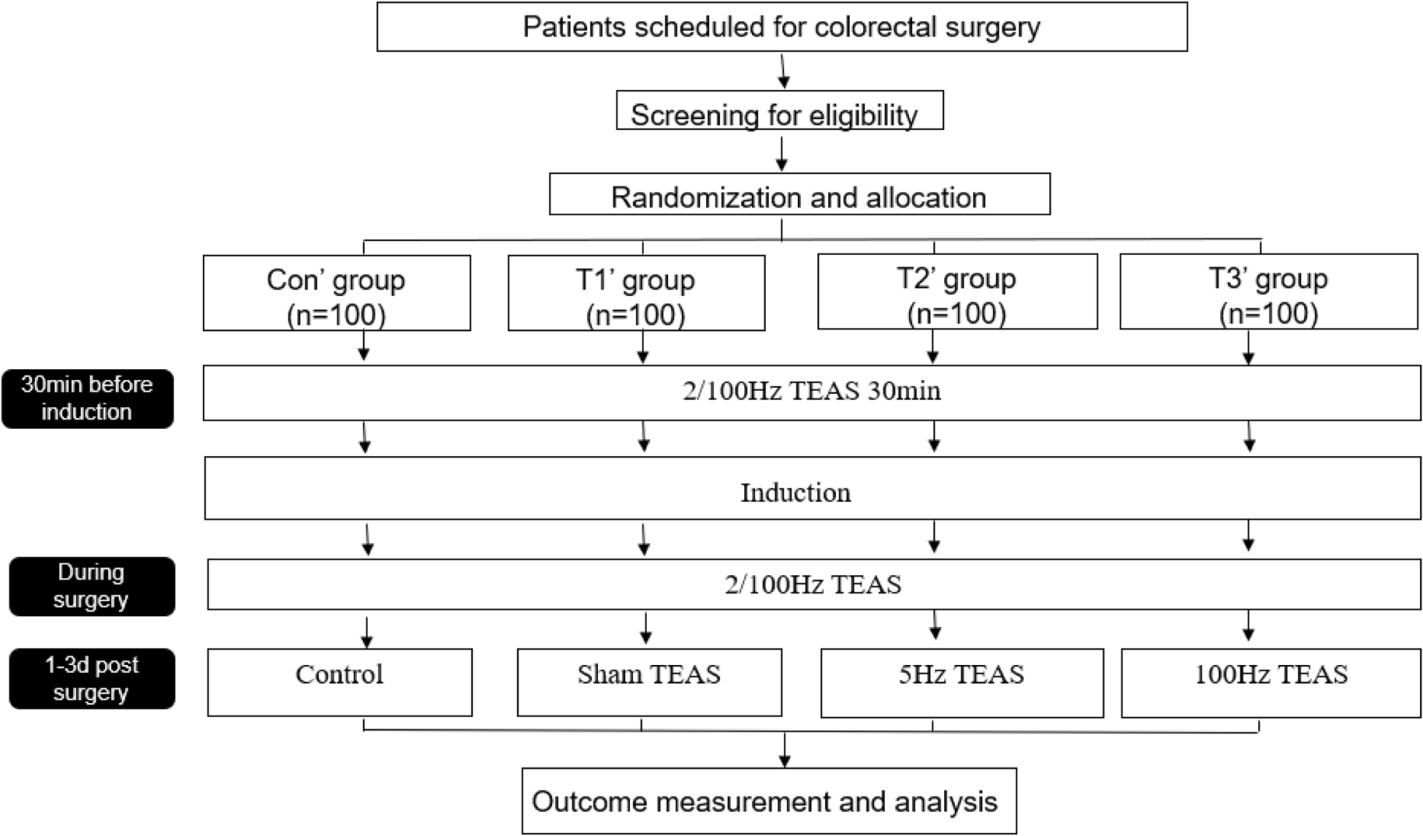
Part 2 study design flowchart. For group details, see Table 2. d days, TEAS transcutaneous electrical acupoint stimulation

Preliminary data analysis based on Part 1 suggest potential benefits of postoperative TEAS for gastrointestinal function recovery, but the effect was slight. Previous animal experiments have suggested that 5 Hz is more effective than 2/100 Hz as a means of promoting the recovery of gastrointestinal function, so we will adjust the study stimulation frequency before initiating Part 2 of this study protocol. In Part 2, all patients will be stimulated at 2/100 Hz (Hegu (LI4) and Neiguan (P6) acupoints; bilateral) before and during surgery. After surgery, patients will receive no TEAS, sham TEAS, 5-Hz TEAS, or 100-Hz TEAS. The same acupoints will be assessed in this trial. In order to observe colorectal function recovery more specifically, Part 2 of this study will focus only on patients undergoing colorectal surgery. The test groups for Part 2 of this study are presented in Table 2, with a study design flowchart shown in Fig. 2.

**Table 2:** Part 2 test groups

Group	Sample size (*n*)	Intervention		
		Preoperative	Intraoperative	Postoperative
Con′	100	2/100 Hz TEAS	2/100 Hz TEAS	Control
T1′	100	2/100 Hz TEAS	2/100 Hz TEAS	Sham TEAS
T2′	100	2/100 Hz TEAS	2/100 Hz TEAS	5 Hz TEAS
T3′	100	2/100 Hz TEAS	2/100 Hz TEAS	100 Hz TEAS

*TEAS* transcutaneous electrical acupoint stimulation

Anesthesia protocols will be identical in Part 1 and Part 2 of this study, with midazolam (0.03 mg/kg), propofol (1.5–2.5 mg/kg), sufentanil (0.3–0.4μg/kg), and rocuronium (0.8 mg/kg) used to achieve effective anesthesia, and with patients undergoing mechanical ventilation after tracheal intubation. Anesthesia maintenance during the intraoperative period will be achieved using remifentanil (0.05–0.2 μg/kg/min), propofol, and rocuronium. Sedation depth will be monitored according to the Bispectral Index (BIS), maintaining BIS values between 45 and 55 by adjusting the propofol infusion rate, and adjusting respiratory parameters to maintain a PetCO_2_ of 35-45 mmHg. If a patient’s heart rate (HR) falls below 50 bpm or to 30% below baseline, they will be given 0.25–0.5 mg atropine; if their HR rises to above 100 bpm or to more than 30% above baseline, esmolol (0.2–0.5 mg/kg) will be given as appropriate. If blood pressure falls to < 90/60 mmHg or to below 30% of baseline, 6 mg ephedrine will be administered to maintain hemodynamic stability. Sufentanil (5–10 μg) or 0.05 mg fentanyl will be given as appropriate following peritoneal suturing, and a prophylactic long-acting 5-HT_3_ receptor antagonist will be used. Patients will be connected with a PCIA pump (250 μg sufentanil plus saline to a total volume of 250 ml; 1 ml/h, 3 ml/bolus, 10-min interval). After skin suturing, sedative and analgesic drug administration will be terminated, and patients will be transferred to a post-anesthesia care unit (PACU). After patients resume spontaneous breathing, muscle relaxation antagonists (neostigmine 1–2 mg, atropine 0.5–1 mg, and flumazenil 0.5 mg, if necessary) will be given, and the extubation time will be recorded. Once the patient is fully awake, pain scores will be assessed and adverse events recorded.

GCP-trained nurses will check the bowel sounds by auscultation of the patients’ left lower abdomen or right lower abdomen at 3:00 a.m., 9:00 a.m., 3:00 p.m., and 9:00 p.m. each day on the 1st, 2nd, and 3rd postoperative days. Each auscultation will last for 3 min.

Patients and their families will be taught how to record postoperative diaries before study initiation, including time of water intake, solid food tolerance, first anal exhaust, and the time at which the patient is first able to leave bed. These diaries will be returned to researchers on the 5th day after operation.

Analgesics will be administered as follows: when the pain NRS score is ≥ 4 even after five consecutive effective bolus applications of the PCIA pump, 50 mg pethidine will be given intramuscularly.

PONV will be treated as follows: nausea levels will be scored via NRS (0–10; 0 for no nausea, 10 for intolerable nausea). 5-HT_3_ receptor antagonists will be given for nausea scores ≥ 7 or when patients vomit more than once. Rescue medication including pethidine and 5-HT_3_ receptor antagonists will be recorded in the remedial tables.

Venous blood (5 ml) will be collected both at the preoperative stage prior to TEAS and 4–5 days after operation. The blood samples will be sent to the Department of Immunology of Peking University Medical Department for immunological and inflammatory index measurements.

When an adverse event occurs, the observer will be able to decide whether to discontinue the observation based on the patient’s condition. If serious adverse events occur, the researchers will immediately take necessary measures to ensure the safety of the patients. All adverse events will be tracked and carefully recorded until the subjects are stable. Those with abnormal laboratory examinations of clinical significance will be monitored until readings return to pretreatment levels.

### Outcome measurement

#### Primary outcome

The primary study outcome will be the time to first bowel sound recovery following surgery, defined as the first time point at which the bowel sounds are heard by GCP-trained nurses at three consecutive time points. The judgment of bowel sounds is based only on the results of auscultation by GCP-trained nurses, even if some patients may feel residual colon gas discharge early after surgery.

#### Secondary outcomes

##### Postoperative recovery of gastrointestinal function

Measured variables for this outcome include: water intake time (the time of first water intake after operation), solid food tolerance time (solid food tolerance indicates that there is no nausea or vomiting within 4 h after a meal), first anal exhaust time, the time at which the patient is able to leave the bed, and the use of gastrointestinal functional adjuvant medicines.

##### Postoperative pain treatment

Recorded variables for this outcome include: the pain intensity score as measured by NRS; analgesia-related side effects; cumulative analgesic drug doses; and the use of PCIA at 9:00 a.m. and 3:00 p.m. on the 1st, 2nd, and 3rd days after operation. Analgesia-related side effects include: respiratory depression, excessive sedation, urinary retention, and pruritus.

##### Acupuncture-balanced anesthesia efficacy

Recorded variables for this outcome include: respiratory and circulatory function during and after operation, depth of anesthesia, dosage of anesthetics and analgesics during induction and intraoperative maintenance, respiratory recovery time after operation, anesthesia recovery time, extubation time, occurrence of restlessness, nausea and vomiting during the recovery period, and NRS pain scores after extubation.

##### Postoperative nausea and vomiting

Recorded variables for this outcome include: the degree of nausea, as scored by NRS (0–10 points). This variable will be scored at 9:00 a.m. and 3:00 p.m. on days 1–5 post operation, and instances of vomiting will also be recorded. 5-HT_3_ receptor antagonists will be given for nausea scores ≥ 7 or when patients vomit ≥ 1 time.

##### Postoperative complications

Recorded variables for this outcome include: postoperative instances of heart, lung, and brain complications.

##### Quality of life assessment

The SF-8 questionnaire will be used to assess quality of life before the operation and 1 month after discharge.

##### Immunological and inflammatory indexes

Immunological and inflammatory indexes include: preoperative and postoperative blood cortisol levels, ACTH, TNF-α, IL-1β, and changes in adaptive immune cells (T cells, B cells, DCs).

We may further examine and analyze these patients’ prognosis.

**Fig. 3:**
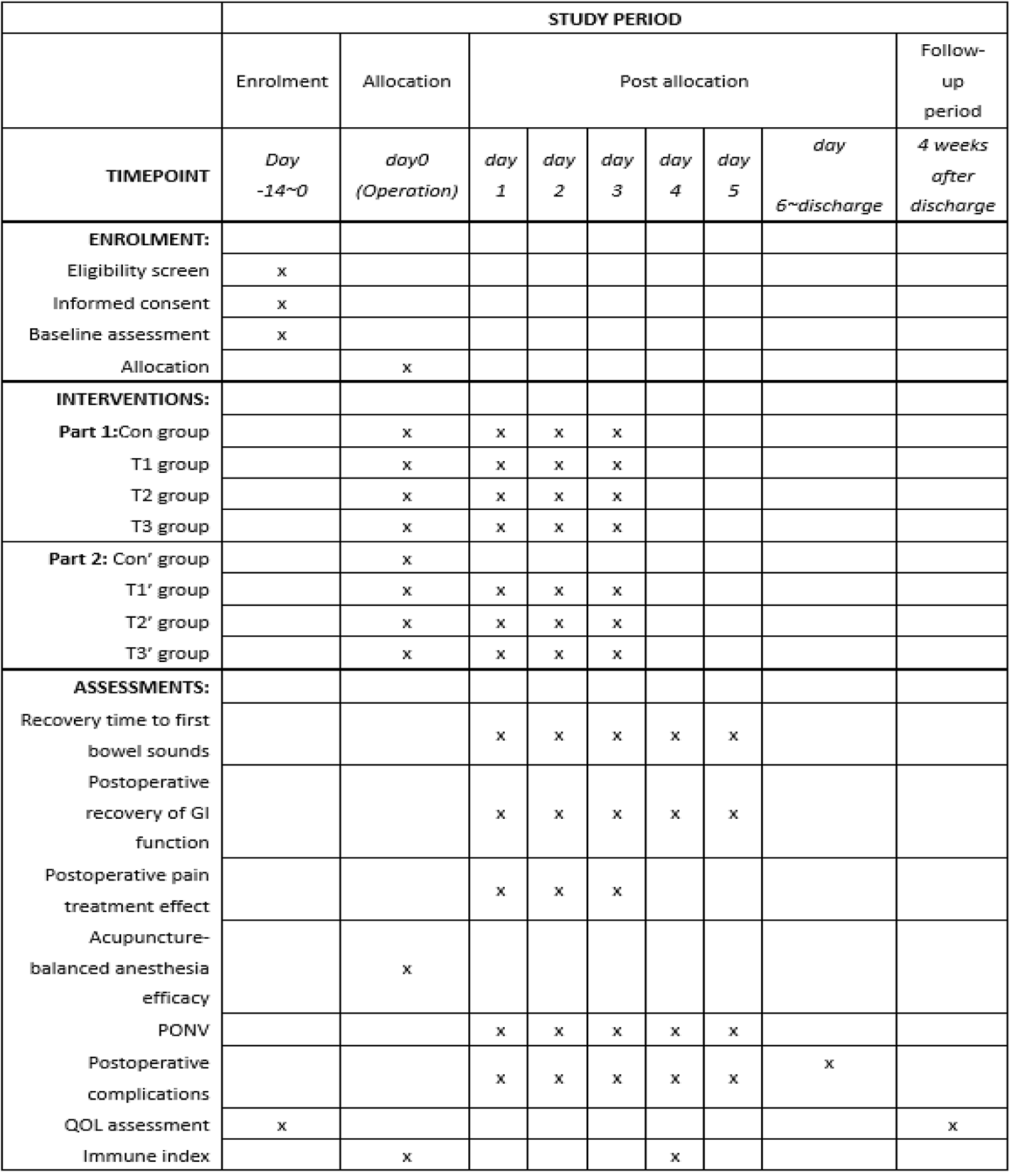
Standard Protocol Items: Recommendations for Interventional Trials (SPIRIT) figure. For group details, see Tables 1 and 2. GI gastrointestinal, PONV postoperative nausea and vomiting, QOL quality of life

The SPIRIT figure is shown in Fig. 3, Additional files 3 and 4.

### Sample size calculations

This study utilizes a superiority design, and the sample size has been calculated according to the primary endpoint of time to first bowel sound recovery following surgery. Based on a single-center preliminary test, this recovery time was 60.320 ± 9.8167 h in sham TEAS-treated individuals and 51.558 ± 17.8499 h in patients who received TEAS during all phases of the perioperative period. Therefore, the difference between the two groups was 8.762 h. Adopting a conservative point of view, the larger standard deviation of 17.8 was used to calculate the optimal sample size. In Part 1, according to a ratio of 3:2 and a power of 90%, a total of 110 and 73 patients were needed per group. To additionally account for a 10% drop-out rate, these case numbers were increased to 120 and 80, respectively. The reason for this unbalanced design between the groups was that both the T1 and T3 groups underwent TEAS before and during the operation, and thus have the potential to achieve a better therapeutic outcome. To ensure maximal possible patient protection, 80 cases were included in the Con and T2 groups, while 120 cases were included in the T1 and T3 groups.

In Part 2, as all patients received the same preoperative and intraoperative interventions, the allocation ratio was altered to 1:1:1:1, with 100 participants per group.

### Statistical analysis

Continuous variables will be expressed as: mean (SD), median (interquartile range (IQR)), or minimum and maximum. For comparisons between treatment groups, analyses of variance (ANOVAs) will be used for normally distributed variables, and the Kruskal–Wallis *H* test will be used for non-normally distributed variables. Categorical variables will be expressed as numbers (%), and will be analyzed via chi-square tests for between-group comparisons.

Statistical significance for this study is defined as α = 0.05 for a two-sided test. *P* values will be rounded to three decimal places; values below 0.001 will be reported as < 0.001. Statistical analyses will be conducted using SAS version 9.4 (SAS Institute Inc.).

### Missing data

Missing primary endpoint data will be imputed using the last-observation-carried-forward (LOCF) method. In cases of missing baseline data, the mean of all patients will be used for imputation. Missing data for other observations will not be imputed.

### Subject disposition

The subject disposition table will be presented for all subjects both overall and arranged according to treatment group. Numbers of study subjects in each population will be summarized, as will numbers who withdraw from the study and their primary reasons for withdrawal.

### Demographic characteristics, vital signs, and medical histories

Demographic characteristics, vital signs, and medical histories for all subjects will be summarized, and between-group comparisons will be conducted as outlined earlier.

### Primary/secondary efficacy endpoints

A covariance model (ANCOVA) will be fitted, with primary endpoint changes from baseline serving as the dependent parameter, and with treatment group, baseline, and study site serving as fixed effects for consideration of site–treatment interactions. For ordinal and categorical variables, a Cochran–Mantel–Haenszel (CMH) test will be used, with study site as a stratifying factor.

### Safety analysis

Safety analyses will be performed in subjects receiving at least one study intervention.

All adverse events will be recorded, and both the numbers of specific adverse events and subjects experiencing adverse events will be quantified as percentages.

All data will be managed by the Peking University Clinical Research Institute, which will be responsible for sample calculations, electronic randomization, data entry, checking, and statistical analyses. Personal information (including sex, age, ID number, BMI, telephone number, past history, etc.) about potential and enrolled participants will be collected in an anonymous way, and will never be leaked to any person or organization.

### Study monitoring

An independent data and safety monitoring committee (DMC) has been formed to monitor the safety of participants in this study. This DMC includes members with expertise clinical practices, statistics, and ethics. Safety reports that include all reported adverse events will be delivered to the DMC every 6 months in a blinded manner. The DMC will be able to recommend whether to stop or to modify the study protocol based on these safety reports. If the incidence of adverse reactions proves unacceptably higher than expected, the DMC has the ability to recommend suspending the study. The DMC can also suspend the study due to the occurrence of any unexpected or serious adverse events.

The first audit will be conducted within 1 week after the first patient enrollment in each research center. Routine follow-up audits will be performed 1–2 times/month, and the frequency of routine audits will be based on the enrollment speed at each center, or any obvious problems at each center (such as overt data abnormalities, program violations, or instances of serious adverse events). The last audit must be completed within 10 working days after the last patient completes follow-up. Auditing steps will include research center personnel training, CRF filling, verification of original data, quality control, screening for failed cases, serious adverse events, and program violations. This process will remain independent from the investigators and the study sponsor.

## Discussion

Previous studies have found that acupuncture can regulate gastrointestinal function through a variety of mechanisms. Acupuncture can promote gastric peristalsis by regulating the vagal nerve [26], inhibiting gastric peristalsis via the somatosensory–nucleus tractus solitarius–rostral ventrolateral medulla–sympathetic efferent pathway [27], thus normalizing biological tissue function. Acupuncture further reduces hyperalgesia through peripheral and central opioid receptors [5], and restores gastrointestinal barrier function by regulating the neuroendocrine immune system and suppressing inflammatory responses [28].

We set the BMI to 18–31 kg/m^2^ for the following reasons: excessive subcutaneous adipose may require much stronger TEAS stimulation intensity; overly thin limbs may make the TEAS electrodes difficult to properly attach, weakening the therapeutic effect; and this trial is focused on a target population of non-obese persons. The challenges of this study are as follows: first, many of the study outcomes must be observed and self-reported by patients, and thus accurate recording of each time point plays an important role in the research results. To mitigate this challenge, researchers will train patients in how to use pain NRS scales and how to evaluate nausea before study initiation. Patients and their families will also be taught how to record times of water intake, solid food tolerance, first anal exhaust, and the time at which the patient was able to leave bed. Second, the strategies for analgesia and reducing PONV must be carefully designed to minimize postoperative discomfort. Third, the adjuvant medicine used must be recorded in as detailed a manner as possible, so as to avoid the interference of other drugs with study results.

## Limitations

There are also limitations to this protocol. The primary outcome will be the time to first bowel sounds recovery following surgery, defined as the first time point at which the bowel sounds are heard by GCP-trained nurses at three consecutive time points. There is a possibility that we may miss the bowel sounds between two auscultations. However, there are clinical trials based on GCP pharmacy supporting this choice of primary endpoint [29, 30], and if all patients are monitored in this way, the result will be comparable and meaningful. The time to first bowel sound recovery is a recognized indicator of the recovery of postoperative bowel function [31]. Previous studies used different methods including patients’ self-report [32] or abdominal auscultation [33, 34] to evaluate this indicator. Among these, patients’ self-report may have memory bias. Abdominal auscultation is more objective, and our protocol will also use auscultation to judge the recovery of first bowel sounds. More importantly, we will record the first time point only when GCP-trained nurses hear the patients’ bowel sounds at three consecutive time points. This can eliminate the interference of transient bowel sounds caused by residual gas in the colon after operation, and make the judgment of the result more accurate.

This study is designed to explore the clinical applications of acupuncture-balanced anesthesia. By observing the role of acupuncture during the preoperative, intraoperative, and postoperative periods associated with abdominal surgery, we will be able to glean new insights into the clinical value and advantages of acupuncture-balanced anesthesia, thereby providing new strategies for improving the prognosis of patients.

## Trial status

This is version 2.0 of the protocol, dated 9 June 2013. Recruitment began on 2 June 2014. Recruitment is anticipated to end on July 2019. Part 1 of this trial has finished participant recruitment and data analysis. Part 2 of this trial is currently recruiting participants.

## Additional files

Additional file 1: Ethics approval. (PDF 108 kb)

Additional file 2: Informed consent form. (PDF 145 kb)

Additional file 3: The SPIRIT checklist. (PDF 156 kb)

Additional file 4: Item No. 2b and item No. 3 of the SPIRIT checklist. (DOC 46 kb)

## Data Availability

The datasets used and/or analyzed during the current study are available from the corresponding author on reasonable request.

## Abbreviations

ANOVA: Analysis of variance
BIS: Bispectral Index
CMH: Cochran–Mantel–Haenszel
CRF: Case report form
DMC: Data and safety monitoring committee
GCP: Good Clinical Practice
HR: Heart rate
IQR: Interquartile range
LOCF: Last observation carried forward
NRS: Numeric Rating Scales
PACU: Post-anesthesia care unit
PCIA: Patient-controlled intravenous analgesia
PONV: Postoperative nausea and vomiting
TEAS: Transcutaneous electrical acupoint stimulation
