# Supplementary material for: The use and protective effects of transcutaneous electrical acupoint stimulation during abdominal surgery: study protocol for a multicenter randomized parallel controlled trial
Source: Trials. 2019 Jul 29;20:462. doi: 10.1186/s13063-019-3558-2 (PMC6664584; doi:10.1186/s13063-019-3558-2)
Supplement: Supplementary file 1 — Ethics approval. (PDF 108 kb) [file 13063_2019_3558_MOESM1_ESM.pdf]

Examination and approval of clinical medicine by Medical Ethics Committee of Peking University People's Hospital

[2013] NO.（09）

<table>
<tr><td>Review project name</td><td colspan="5">Acupuncture balanced anesthesia used in abdominal operation and its protective effects</td></tr>
<tr><td rowspan="3">Application Department</td><td rowspan="3" colspan="2">Department of Anesthesiology</td><td colspan="2">Project Leader: Yi Feng</td><td>Title: Professor</td></tr>
<tr><td colspan="2">Main participant: Qi Yan</td><td>Title: Resident</td></tr>
<tr><td colspan="2">Head of Department：Yi Feng</td><td>Title: Professor</td></tr>
<tr><td colspan="6">Funding：973 project</td></tr>
<tr><td>Declaration document</td><td colspan="2">1. Application Form for Ethical Review
2. Research Programmes
3. Informed Consent Form (Version 1.0)</td><td>Participating units</td><td colspan="2">Five Units, including Peking University Third Hospital</td></tr>
<tr><td colspan="3">Meeting time: 14 May 2013</td><td colspan="3">Meeting Venue: Conference Room 15, Peking University People's Hospital</td></tr>
<tr><td rowspan="3">Comments of the Ethics Committee</td><td>Agree</td><td>Consent after necessary modification</td><td>Disagree</td><td colspan="2">Revision and retrial</td></tr>
<tr><td></td><td>√</td><td></td><td colspan="2"></td></tr>
<tr><td colspan="5">To amend the Informed Consent Form:
1. Unify the name of "operation item";
2. Detailed introduction of "operation steps";
3. Explain "randomness" and suggest optimizing "group name".
4. Inform the blood sampling test items in detail;
5. Individual "About Fees" and clearly inform free items;
6. Supplementary adverse reactions and treatment measures of the tested products;
7. To supplement this test, it is necessary to follow the Regulations on Clinical Trials of Medical Devices.
8. Correct text errors.

Signature by the Chairman (Vice-Chairman):

Date:</td></tr>
</table>

北京大学人民医院医学伦理委员会临床医学审评批件

[2013] 院伦审临医字第（09）号

<table>
<tr><td>评审项目名称</td><td colspan="5">针药复合麻醉在腹部手术的应用及机体保护效应</td></tr>
<tr><td rowspan="3">申请科室</td><td rowspan="3" colspan="2">麻醉科</td><td colspan="2">项目负责人：冯 艺</td><td>职 称：教 授</td></tr>
<tr><td colspan="2">主要参加者：闫 琦</td><td>职 称：住院医师</td></tr>
<tr><td colspan="2">科室负责人：冯 艺</td><td>职 称：教 授</td></tr>
<tr><td colspan="6">课题来源：973</td></tr>
<tr><td>申报文件</td><td colspan="2">1. 伦理审查申请表
2. 研究方案
3. 知情同意书（Version 1.0）</td><td>参加单位</td><td colspan="2">北京大学第三医院等 5 家单位</td></tr>
<tr><td colspan="3">会议时间：2013 年 5 月 14 日</td><td colspan="3">会议地点：北京大学人民医院第 15 会议室</td></tr>
<tr><td rowspan="3">伦理委员会审评意见</td><td>同 意</td><td>作必要修改后同意</td><td>不同意</td><td colspan="2">修改后重审</td></tr>
<tr><td></td><td>√</td><td></td><td colspan="2"></td></tr>
<tr><td colspan="5">修改知情同意书：
1. 统一“操作项目”名称；
2. 详细介绍“操作步骤”；
3. 解释“随机”，建议优化“分组名称”；
4. 详细告知取血检测项目；
5. “关于费用”单列，明确告知免费项目；
6. 补充受试产品的不良反应及处理措施；
7. 补充此试验尚需遵循《医疗器械临床试验规定》；
8. 修改文字错误。

主任委员（副主任委员）签字：[illegible]
2013 年 5 月 17 日</td></tr>
</table>

Medical Ethics Committee of Peking University People's Hospital

Reply Letter to the " Consent after modification " Project from the Ethics Committee

After re-examination by the Medical Ethics Committee of our hospital, Professor Yi Feng, the project leader, revised the Informed Consent Form(Version 1.0) of the project《Acupuncture balanced anesthesia used in abdominal operation and its protective effects》according to the opinions of the Ethics Committee on May 14, 2013. The revised Informed Consent Form (Version 2.0) meets the requirements of the Ethics Committee.

Agree to start clinical research.

Chairman:

Date:

北京大学人民医院医学伦理委员会

"修改后同意"项目伦理审查回复函

经我院医学伦理委员会再次审查，项目负责人冯艺教授根据2013年5月14日伦理委员会审查意见，对《针药复合麻醉在腹部手术的应用及机体保护效应》项目的知情同意书（Version 1.0）进行了修改，修改后的知情同意书（Version 2.0）符合伦理委员会要求。

同意开始临床研究。

主任委员：[illegible]

[illegible]年[illegible]月[illegible]日
