# Supplementary material for: The use and protective effects of transcutaneous electrical acupoint stimulation during abdominal surgery: study protocol for a multicenter randomized parallel controlled trial
Source: Trials. 2019 Jul 29;20:462. doi: 10.1186/s13063-019-3558-2 (PMC6664584; doi:10.1186/s13063-019-3558-2)
Supplement: Supplementary file 2 — Informed consent form. (PDF 145 kb) [file 13063_2019_3558_MOESM2_ESM.pdf]

针药复合麻醉在腹部手术的应用及机体保护效应多中心，随机，对照，盲法临床研究

知情同意书

Informed Consent Form

知情同意书版本号：Version 2.0

临床研究单位：________________

主要研究者：________________

联系电话：________________

针药复合麻醉在腹部手术的应用及机体保护效应受试者知情同意书

我已收到并详细阅读了针药复合麻醉在腹部手术的应用及机体保护效应的多中心，随机，对照，盲法的临床研究的受试者须知。我已了解这是一项临床试验，我的医生已经向我详细介绍了该临床试验的目的、方法、可能出现的不良反应以及我的风险和受益，并对有关问题给予了解答。我的医生还向我详细解释了本次试验我需要进行的配合。我在充分了解须知全部内容以及参加试验可能带来的利弊后，自愿参加并认真完成本试验。我理解：

1. 尽管研究记录被保密，但各级食品药品监督管理部门、伦理委员会及申办者指派的稽查员和监查人员都有可能接触到我的个人资料，以上人员有责任为我的个人情况保密。
2. 我是本研究的受试者，我将遵守本研究方案和受试者须知的要求，完成本试验。
3. 如果在试验过程中出现任何不良事件，我都会立即通知我的医生。
4. 在研究过程的任何阶段，我有权退出，而不会受到歧视和报复。
5. 我理解参加此项研究可能的受益和风险，我愿意为医学科学的进步做出奉献。
6. 我自愿参加本试验，并与研究人员充分合作。
7. 我将获得一份由双方签名并注明日期的知情同意书副本。

受试者签名______________　　　日期________年____月____日

我已给予受试者足够的时间就受试者须知、知情同意书和试验方案等提出问题，并尽我所能给予了回答。

研究者签名______________　　　日期________年____月____日

联系方式：电话：______________________________________

地址：______________________________________

Acupuncture balanced anesthesia used in abdominal operation and its protective effects: A multicenter, randomized, controlled, blind clinical study

Informed Consent Form

Version Number of Informed Consent: Version 2.0

Clinical Research Unit: ____________________________

Major Researcher: ____________________________

Telephone number.: ____________________________

Acupuncture balanced anesthesia used in abdominal operation and its protective effects

Informed Consent Form

I have received and read in detail the instructions for participants in a multicenter, randomized, controlled, blind clinical study of the application of combined acupuncture and drug anesthesia in abdominal surgery and its protective effects on the body. I have learned that this is a clinical trial, and my doctor has given me a detailed description of the purpose, method, possible adverse reactions, and my risks and benefits of the clinical trial, and answered the relevant questions. My doctor also explained to me in detail the cooperation I needed in this experiment. After fully understanding the contents of the instructions and the pros and cons of participating in the experiment, I volunteered to participate in and conscientiously completed the experiment. I understand:

1. Although the research records are kept confidential, the inspectors and inspectors appointed by food and drug regulatory authorities, ethics committees and bidders at all levels may have access to my personal data. The above persons are responsible for keeping my personal information confidential.
2. I am the subject of this study. I will complete this experiment in accordance with the requirements of the research program and the participants' instructions.
3. If any adverse events occur during the experiment, I will inform my doctor immediately.
4. At any stage of the research process, I have the right to withdraw without discrimination or retaliation.
5. I understand the potential benefits and risks of participating in this research. I am willing to contribute to the progress of medical science.
6. I volunteered to participate in the experiment and cooperated fully with the researchers.
7. I will get a copy of the informed consent signed and dated by both parties.

Subject signature______________ Date___________

I have given the subjects enough time to ask questions about the subject's instructions, informed consent and test plan, and to answer their questions to the best of my ability.

Researcher's signature___________ Date___________

Contact information： Tel： ______________________________

Address: _______________________________
