# Supplementary material for: The use and protective effects of transcutaneous electrical acupoint stimulation during abdominal surgery: study protocol for a multicenter randomized parallel controlled trial
Source: Trials. 2019 Jul 29;20:462. doi: 10.1186/s13063-019-3558-2 (PMC6664584; doi:10.1186/s13063-019-3558-2)
Supplement: Supplementary file 4 — Item No. 2b and item No. 3 of the SPIRIT checklist. (DOC 46 kb) [file 13063_2019_3558_MOESM4_ESM.doc]

**2b: All items from the World Health Organization Trial Registration Data Set.**

| **Data category** | **Information** |
| --- | --- |
| Primary registry and trial identifying number | Chinese Clinical Trial Registry. ChiCTR-TRC-14004435. |
| Date of registration in primary registry | 26 March 2014 |
| Secondary identifying numbers | NA |
| Source(s) of monetary or material support | The National Basic Research Program of China (973 project) No. 2013CB531900 |
| Primary sponsor | NA |
| Secondary sponsor(s) | NA |
| Contact for public queries | Yi Feng, MD, PhD; E-mail: doctor_yifeng@sina.com |
| Contact for scientific queries | Yi Feng, MD, PhD  Peking University People’s Hospital, China |
| Public title | The use and protective effects of transcutaneous electrical acupoint stimulation during abdominal surgery |
| Scientific title | Acupuncture balanced anesthesia used in abdominal operation and its protective effects |
| Countries of recruitment | China |
| Health condition(s) or problem(s) studied | Transcutaneous electrical acupoint stimulation (TEAS), anesthesia management, gastrointestinal surgery, organ protection |
| Intervention(s) | TEAS at different perioperative phases;  TEAS at different frequencies; |
| Key inclusion and exclusion criteria | Inclusion criteria: Aged 18-75 years old; Undergoing elective abdominal surgery; Body mass index 18-31 kg/m^2^; American Society of Anesthesiologists grading I-III; Informed consent signed.  Key exclusion criteria: Scars, sensory impairments, infections around the acupoints of interest; Peripheral nerve injury, mental or neurological diseases; History of spinal surgery; Participated in other clinical trials within the past four weeks; Cannot understand Numeric Rating Scales (NRS) scores, or reject Patient Controlled Intravenous Analgesia(PCIA); Patients with preoperative pain or a history of analgesic usage; Patients who require an intraoperative colostomy, or return to the intensive care unit. |
| Study type | Multi-center, Randomized, Parallel controlled trial |
| Date of first enrolment | June 2014 |
| Target sample size | 800 |
| Recruitment status | Recruiting |
| Primary outcome | The recovery time to first bowel sounds |
| Key secondary outcomes | Postoperative recovery of gastrointestinal function; Postoperative pain treatment; Acupuncture-balanced anesthesia efficacy; Postoperative nausea and vomiting; Postoperative complications; Quality of life assessment. |

**3. Date and version identifier**

Revision Chronology:

| 2013-May-14: | Original |
| --- | --- |
| 2013-June-9: | Amendment reason: At the request of Ethics Committee, we unified the name of "operation item"，detailed introduction of "operation steps"，explained "randomness"，informed the blood sampling test items in detail，supplemented adverse reactions and treatment measures of the tested products. |
